# Regulation of CcpA on the growth and organic acid production characteristics of ruminal *Streptococcus bovis* at different pH

**DOI:** 10.1186/s12866-021-02404-x

**Published:** 2021-12-15

**Authors:** Yaqian Jin, Chao Wang, Yaotian Fan, Mawda Elmhadi, Ying Zhang, Hongrong Wang

**Affiliations:** 1 Laboratory of Metabolic Manipulation of Herbivorous Animal Nutrition, College of Animal Science and Technology, Yangzhou University, Yangzhou, 225009 People’s Republic of China; 2 School of Biomedical Sciences, The University of Western Australia, M Block, Queen Elizabeth II Medical Centre, Nedlands, WA 6009 Australia

**Keywords:** *Streptococcus bovis*, Catabolite control protein A (CcpA), pH, Organic acids, Fermentation pattern

## Abstract

**Background:**

Catabolite control protein A (CcpA) regulates the transcription of lactate dehydrogenase and pyruvate formate-lyase in *Streptococcus bovis*, but knowledge of its role in response to different pH is still limited. In this study, a *ccpA*-knockout strain of *S. bovis* S1 was constructed and then used to examine the effects of *ccpA* gene deletion on the growth and fermentation characteristics of *S. bovis* S1 at pH 5.5 or 6.5.

**Results:**

There was a significant interaction between strain and pH for the maximum specific growth rate (μ_max_) and growth lag period (λ), which caused a lowest μ_max_ and a longest λ in *ccpA*-knockout strain at pH 5.5. Deletion of *ccpA* decreased the concentration and molar percentage of lactic acid, while increased those of formic acid. Strains at pH 5.5 had decreased concentrations of lactic acid and formic acid compared to pH 6.5. The significant interaction between strain and pH caused the highest production of total organic acids and acetic acid in *ccpA*-knockout strain at pH 6.5. The activities of α-amylase and lactate dehydrogenase decreased in *ccpA*-knockout strain compared to the wild-type strain, and increased at pH 5.5 compared to pH 6.5. There was a significant interaction between strain and pH for the activity of acetate kinase, which was the highest in the *ccpA*-knockout strain at pH 6.5. The expression of pyruvate formate-lyase and acetate kinase was higher in the *ccpA*-knockout strain compared to wild-type strain. The lower pH improved the relative expression of pyruvate formate-lyase, while had no effect on the relative expression of acetate kinase*.* The strain × pH interaction was significant for the relative expression of lactate dehydrogenase and α-amylase, both of which were highest in the wild-type strain at pH 5.5 and lowest in the *ccpA*-knockout strain at pH 6.5.

**Conclusions:**

Overall, low pH inhibited the growth of *S. bovis* S1, but did not affect the fermentation pattern. CcpA regulated *S. bovis* S1 growth and organic acid fermentation pattern. Moreover, there seemed to be an interaction effect between pH and *ccpA* deletion on regulating the growth and organic acids production of *S. bovis* S1.

**Supplementary Information:**

The online version contains supplementary material available at 10.1186/s12866-021-02404-x.

## Introduction

Generally, high-concentrate diets based on grains are applied in modern intensive ruminant production to increase production. However, ruminants fed on high-concentrate diets display increased yields of volatile fatty acids (VFA) and lactic acid in the rumen and decreased rumen pH, which may cause subacute ruminal acidosis [1, 2]. In this process, the concentration of lactic acid is much lower than VFA because there is a balance between the production of lactic acid and its conversion into VFA. Once the balance is broken, lactic acid begins to accumulate in the rumen. Because lactic acid is a stronger acid than VFA (pKa 3.9 vs 4.7–4.9), its accumulation often brings about a downward spiral in ruminal pH, which may induce rumen lactic acidosis [3]. Previous studies demonstrated that an initial overgrowth of *Streptococcus bovis* is the major cause of increasing lactic acid and declining ruminal pH, which will result in the inability of most ruminal bacteria, and the acid-tolerant *Lactobacilli* becoming predominant [4, 5]. Therefore, *S. bovis* is considered the major etiologic agent of rumen lactic acidosis, and suppressing its overgrowth and overproduction of lactic acid is vital to prevent rumen lactic acidosis when animals are supplied with a high grain diet.

*S. bovis* is a gram-positive bacterium that utilizes starch and soluble sugars as substrate in the rumen, thereby producing lactic acid, formic acid, acetic acid and ethanol. Several previous studies have shown that the fermentation products of *S. bovis* are regulated by the pH, carbon source and substrate concentration [6–10]. Our previous work with *S. bovis* S1 demonstrated that the carbohydrate source (soluble starch vs. glucose) had a major effect on lactic acid production due to the transcriptional regulation of metabolic genes [10]. Furthermore, we evaluated the relative importance of pH and starch concentration in fermentation characteristics of *S. bovis* S1 and found that the fermentation of the strain was more sensitive to the pH changes [11]. In addition to the environmental factors, the fermentation pattern of *S. bovis* is also controlled by the catabolite control protein A (CcpA) [12], which is a pleiotropic regulatory protein in low-GC gram-positive bacteria involved in carbon and nitrogen metabolism, biofilm formation, toxic gene expression, and other physiological processes. Several studies have reported that CcpA activates or inhibits transcriptional expression of the target gene via binding to the specific sequence of its target gene, such as catabolite response element (*cre*) [13–15]. The regulation of target genes by CcpA is not only associated with heat-stable protein (HPr) [16–18] and small molecule compounds such as fructose-1,6-diphosphate (FDP) and 6-phosphate glucose [19], but also affected by the environmental factors, such as substrate type, oxygen presence and pH [20–22]. Based on the above literatures and our previous work, we hypothesized that CcpA transcriptionally regulates the fermentation pattern of *S. bovis* S1, which might be is affected by pH. To test this hypothesis, we constructed the *ccpA* gene knockout mutant of *S. bovis* S1 using homologous recombination technology, and used it to investigate the effect of *ccpA* gene deletion on the growth and fermentation characteristics of *S. bovis* S1 at different pH.

## Materials and methods

### Bacterial strains

Strain *S. bovis* S1 (CCTCC AB 2016240) used in this study was previously isolated from the rumen fluid of Saanen dairy goats (late lactation) in our laboratory [10]. Its *ccpA*-disrupted mutant was constructed by homologous recombination as follows. First, DNA fragments corresponding to the upstream (1053 bp fragment; primer pairs *ccpA* Up F/*ccpA* Up R, Table 1) and downstream (1101 bp fragment; primer pairs *ccpA* Down F/*ccpA* Down R, Table 1) sequences of *ccpA* were amplified by PCR using *S. bovis* S1 genomic DNA as a template. The erythromycin resistance gene *erm* was amplified with the primers *erm* F and *erm* R (Table 1). The PCR product was purified using a PCR purification kit (Qiagen, Beijing, China) according to the manufacturer’s instructions. The amplified fragments were respectively cloned into EcoRI, BamHI and SacI restriction sites of pUC19 vector to generate pUC19-*ccpA* up-*erm*-*ccpA* down (pCE). The recombinant vector pCE was electroporated into *S. bovis* S1 cells using an electroporation system at 2.5 kV, 200 Ω, and 25 μF. Finally, knockout mutants were selected on MRS plates containing 1 μg/mL erythromycin at 37 °C for 3–4 days.

**Table 1:** Primers used in this study

Primer names	Primer sequences (5′-3′)	Purpose of use	Product length (bp)	Reference or source
*ccpA* up-F	TGTAAAACGACGGCCAGTGAATTCGTTCCAAGGTCAAACAAAAGTAGAG	Construction of *ccpA*-knockout mutant	1053	This study
*ccpA* up-R	AAGCTGTCAAACATGAGAATTAGAGCTCTATTGGACTTCCTTTCTATTTG			
*ccpA* down-F	AGCTTTTGCTAAAGAAGAATTGGATCCTTTCCAAAAAGGATACTATGAC	Construction of *ccpA*-knockout mutant	1101	This study
*ccpA* down-R	CAAGCTTGCATGCCTGCAGGTCGACGCAACTTTATCAATGCTACGAC			
*erm*-F	CAAATAGAAAGGAAGTCCAATAGAGCTCTAATTCTCATGTTTGACAGCTT	Construction of *ccpA*-knockout mutant	1207	This study
*erm*-R	GTCATAGTATCCTTTTTGGAAAGGATCCAATTCTTCTTTAGCAAAAGCT			
*ccpA 1*-F	TGGTGAATCATTACTTGTAAGA	Verification of *ccpA*-knockout mutant	426	This study
*ccpA 1*-R	GAGTTCTCTCGCTCACGCACAC			
*ccpA 2*-F	AAACCTTCTTTCTAATTACCCC	Verification of *ccpA*-knockout mutant	1827	This study
*ccpA 2*-R	GGCATAAATCGGCTTGTCAACG			
*16S*-F	GAACACCGGTGGCGA	RT-qPCR		Asanuma et al. [23]
*16S*-R	CTCATCGTTTACGGCG			
*pfl*-F	GGTTACATCTACGACTACGA	RT-qPCR	119	Chen et al. [10]
*pfl*-R	TGGCTACGAAGACGAGTA			
*ldh*-F	GGTTCTTCTTACGCATTCG	RT-qPCR	190	Chen et al. [10]
*ldh*-R	TAACTACAAGGTCAGCATCT			
*α-amy*-F	TCAAGCACTGGAA TCAACTA	RT-qPCR	109	Chen et al. [10]
*α-amy*-R	GCCGTAA TAA TCTCCGTAGA			
*ack*-F	TGGCCAGAAACAGTCGGAAA	RT-qPCR	134	This study
*ack*-R	CTACCACACGGTGACCAACA			

The result of knockout was validated by PCR and DNA sequencing. Briefly, genomic DNA of *S. bovis* S1 wild-type strain and its mutant strain were extracted with the TIANamp Bacteria DNA Kit (DP302, Tiangen Biotech Co. Ltd., Beijing, China) and used as the templates. Primers used for verification are shown in Table 1, which were designed based on the internal sequence of the *ccpA* gene (*ccpA1*-F/*ccpA1*-R) or across the upstream and downstream sequences of the *ccpA* gene (*ccpA2*-F/*ccpA2*-R). PCR analysis was performed using the 2 × Taq PCR MasterMix II Kit (KT211–02, Tiangen Biotech Co. Ltd., Beijing, China) according to the manufacturer’s instructions. The amplified PCR products were electrophoresed on a 1% (wt/vol) agarose gel. Sequence analysis for wt2 and ko2, which were the products of primer *ccpA2* amplified from *S. bovis* S1 wild-type strain and the *ccpA*-knockout strain respectively, was performed by Shanghai Sangon Biological Engineering Technology and Services Co., Ltd. for further verification.

### Experimental design

The experiment treatments were arranged as a 2 × 2 factorial design: two strains, which were *S. bovis* S1 wild-type strain and its *ccpA*-disrupted mutant respectively, were cultured in the media at pH 5.5 or pH 6.5. Specific operations are as follows. First, the seed cultures of *S. bovis* S1 wild-type strain and its mutant strain were cultured in a modified MRS medium [10] in an anaerobic workstation (DG250, Don Whitley Scientific, England) at 37 °C. Next, the seed cultures of both strains (at exponential phase) were transferred with 1% (v/v) inoculum into 200 mL anaerobic serum bottles containing 100 mL basal medium, respectively. The basal medium was prepared according to Chen et al. [10], and it contained: 0.45 g/L KH_2_PO_4_, 0.45 g/L K_2_HPO_4_, 0.9 g/L NaCl, 0.9 g/L (NH_4_)_2_SO_4_, 0.12 g/L CaCl_2_·2H_2_O, 0.19 g/L MgSO_4_·7H_2_O, 1.0 g/L tryptone, 1.0 g/L yeast extract, 0.6 g/L cysteine hydrochloride, and 3.0 g/L soluble starch (Cat#G8300, Solarbio, Beijing, China). The original pH of the medium was 4.2, which was adjusted to 5.5 and 6.5 using 10% NaOH (w/v) before sterilization. The cultivation was performed in an anaerobic thermostat shaker at 37 °C and 160 rpm, and the pH of the medium was monitored using a pH meter (SevenExcellence-S470, Mettler-Toledo, Switzerland) and adjusted to 5.5 or 6.5 by continuous titration with 10% NaOH (w/v) using an injection pump (TYD02–10, Leadfluid Technology Co., Ltd., Baoding, China). Three replicates were set for each treatment.

### Sample collection

Cell growth were monitored by measuring OD values at 600 nm every hour using SpectraMax M5 plate reader (Molecular Devices Corporation, USA). The cultures of each sample were harvested in duplicate when they reached the exponential growth phase. A portion of the samples was quickly placed in liquid nitrogen and stored at − 80 °C for enzyme activity assay, while the remaining samples were centrifuged at 13,400×g for 2 min at 4 °C. The obtained cell pellets were quickly frozen in liquid nitrogen for 15 min, then stored at − 80 °C for further RNA isolation; the supernatant was filtered using a 0.22 μm filter membrane and stored at − 80 °C for the determination of organic acids.

### Determination of organic acids and enzyme activity

A high performance liquid chromatographer (HPLC, Shimadzu, Japan) equipped with an acclaim OA column (Sepax Carbomix H-NP) and a UV detector was used to determine concentrations of organic acids (lactic acid, formic acid, and acetic acid) in the supernatant. The column temperature was kept at 55 °C, and the mobile phase was 2.5 mM H_2_SO_4_ with the flow rate set at 0.5 mL/min. Organic acids were then measured with a UV detector set at 210 nm. Commercial assay kits (Comin Biotechnology Co., Ltd., Suzhou, China) were used to measure the activities of α-amylase (Cat. No. DFMA-1-Y), lactate dehydrogenase (Cat. No. LDH-1-Y), and acetate kinase (Cat. No. ACK-1-Y). Before the measurement, 2 mL of bacterial fluid was centrifuged at 8000×g for 10 min at 4 °C and the cell pellets were resuspended in the extracting solution from the kit. To lyse the bacterial cells, the suspension was then sonicated on an ice bath using a VCX-130 Sonicator (Sonics, USA) for 10 min with 30 s pulse on and 30 s pulse off, at 100 W. Unbroken cells were removed by centrifugation at 8000×g for 10 min at 4 °C, and the supernatant was collected for the determination of enzyme activities according to the corresponding kit instructions.

### RNA extraction and RT-qPCR analysis

Before RNA extraction, 200 μL of bacterial fluid was centrifuged at 13,400×g for 2 min at 4 °C and the cell pellets were incubated with 180 μL of lysozyme solution (3 mg/mL) at room temperature for 10 min to break the cell walls. Total RNA was extracted from *S. bovis* S1 wild-type and mutant strains using the RNAprep Pure Bacteria Kit (DP430, Tiangen Biotech Co. Ltd., Beijing, China) according to the manufacturer’s instructions. The quantity and quality of total RNA were evaluated using Agilent 2100 Bioanalyzer (Agilent Technologies, Palo Alto, CA, USA) and agarose gel electrophoresis, respectively. Next, total RNA was reverse-transcribed to cDNA in a 20 μL reaction mixture using the Primer-Script™ Reagent Kit (TaKaRa Biotechnology Co. Ltd., Dalian, China) according to the manufacturer’s instructions. The reverse transcriptase quantitative PCR (RT-qPCR) was performed with the ABI Step-One-Plus RT-PCR system (ABI 7500, Applied Biosystems, Foster City, CA) using the TB Green Premix Ex Taq™ II Kit (TaKaRa Biotechnology Co. Ltd., Dalian, China). The reaction was first performed in a 20 μL reaction solution containing 10 μL 2 × TB Green Premix Ex Taq II, 1.6 μL primer, 1 μL cDNA, 0.4 μL 50 × ROX, and 7.0 μL RNase-free water. The RT-qPCR conditions were as follows: 95 °C for 30 s, followed by 40 cycles of the amplification at 95 °C for 5 s and 60 °C for 34 s. Primers of *α-amy* (gene encoding α-amylase), *ldh* (gene encoding lactate dehydrogenase), *pfl* (gene encoding pyruvate formate lyase), and *ack* (gene encoding acetate kinase) were designed using Primer-Blast and the *16S* rRNA was used as an internal reference gene (Table 1). Relative gene expression was normalized to expression of the *16S* rRNA gene and calculated using the 2^-△△CT^ method.

### Data analysis

Results were expressed as means and SEMs. All data were analyzed as a 2 × 2 factorial design by ANOVA using GLM procedures of SPSS (SPSS 25.0, IBM, USA) software. The statistical model included the effects of strain (wild-type strain or *ccpA*-knockout strain) and pH (6.5 or 5.5), as well as their interaction. Graphs were drawn using GraphPad Prism 8. The logistic function model [24] was used to non-linearly fit the growth curves of bacteria. The specific model is: “Ln(y_t_/y_0_) = D/(1 + exp.((4 × μ_max_/D)(λ-t) + 2))”, where y_t_ is OD value at time t, y_0_ is initial OD value, t is hour of the incubation, μ_max_ is the maximum specific growth rate, D is the limit of ln (y_t_/y_0_), and λ is the growth lag period (h).

## Results

### Verification of *ccpA* gene knockout

**Fig. 1:**
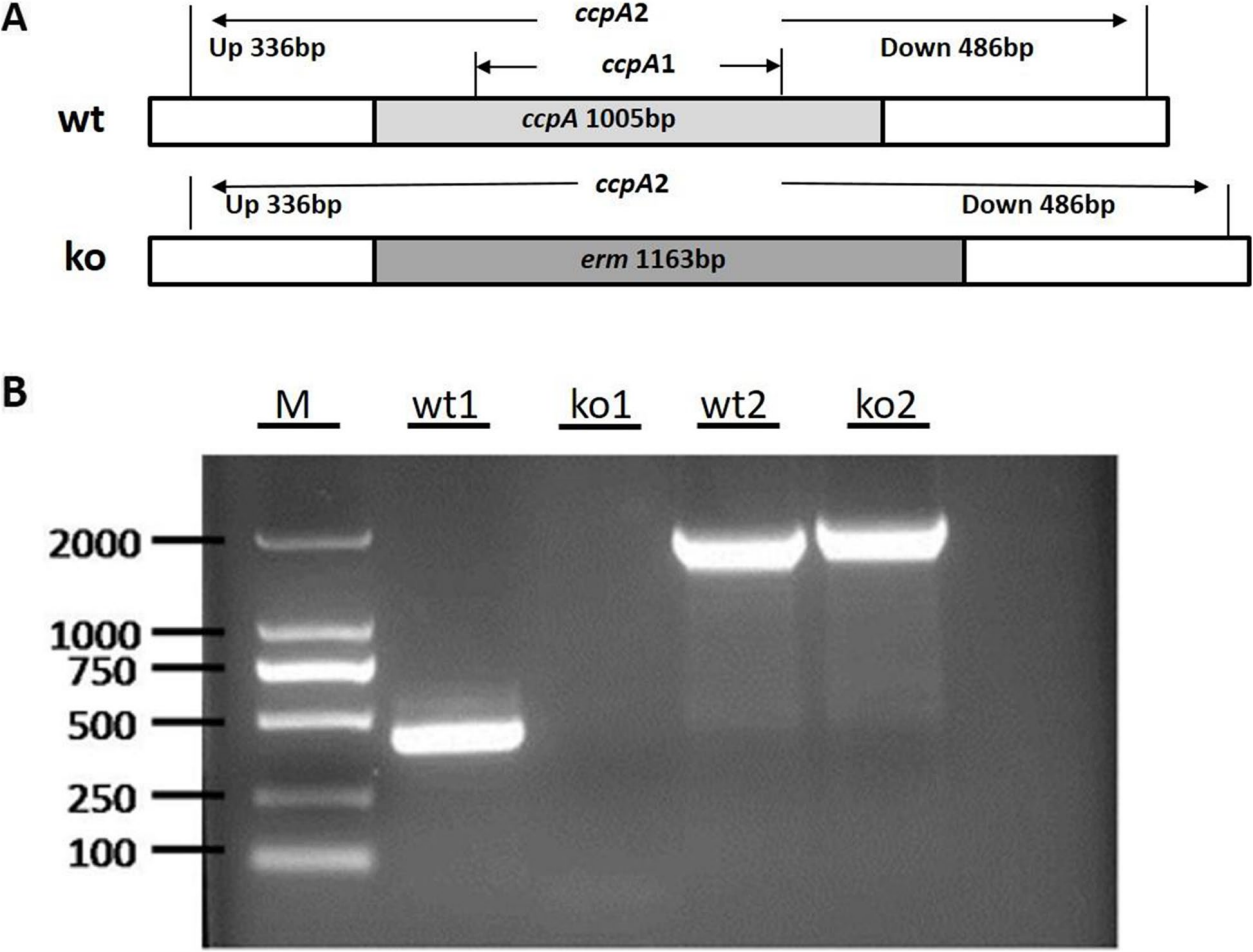
The result of PCR verification for wild-type strain (wt) and *ccpA*-knockout strain (ko) of *S. bovis* S1. **A** Diagram of PCR verification for genomic structure of wild-type strain (wt) and *ccpA*-knockout strain (ko). *ccpA 1* was designed based on the internal sequence of the *ccpA* gene, with a size of about 426 bp; *ccpA 2* was designed across the upstream and downstream sequences of the *ccpA* gene, with a size of about 1827 bp for wild-type strain and 1985 bp for *ccpA*-knockout strain. **B** The result of the PCR verification. wt1: a 426 bp PCR product of *ccpA 1* amplified in wild-type strain; ko1: no PCR product of *ccpA 1* amplified in *ccpA*-knockout strain; wt2: a 1827 bp PCR product of *ccpA 2* amplified in wild-type strain; ko2: a 1985 bp PCR product of *ccpA 2* amplified in *ccpA*-knockout strain

The results of PCR verification of *ccpA* gene knockout are shown in Fig. 1. Figure 1A shows the expected sizes and targets of the PCR products, which was confirmed by the results of gel electrophoresis as shown in Fig. 1B (the full-length picture is provided in the Supplementary file). These results indicated that the *ccpA* gene was deleted successfully in the *ccpA*-knockout strain. In addition, the DNA sequencing results of wt2 and ko2 further confirmed that the *ccpA* deletion strain was constructed.

### Growth characteristics

**Fig. 2:**
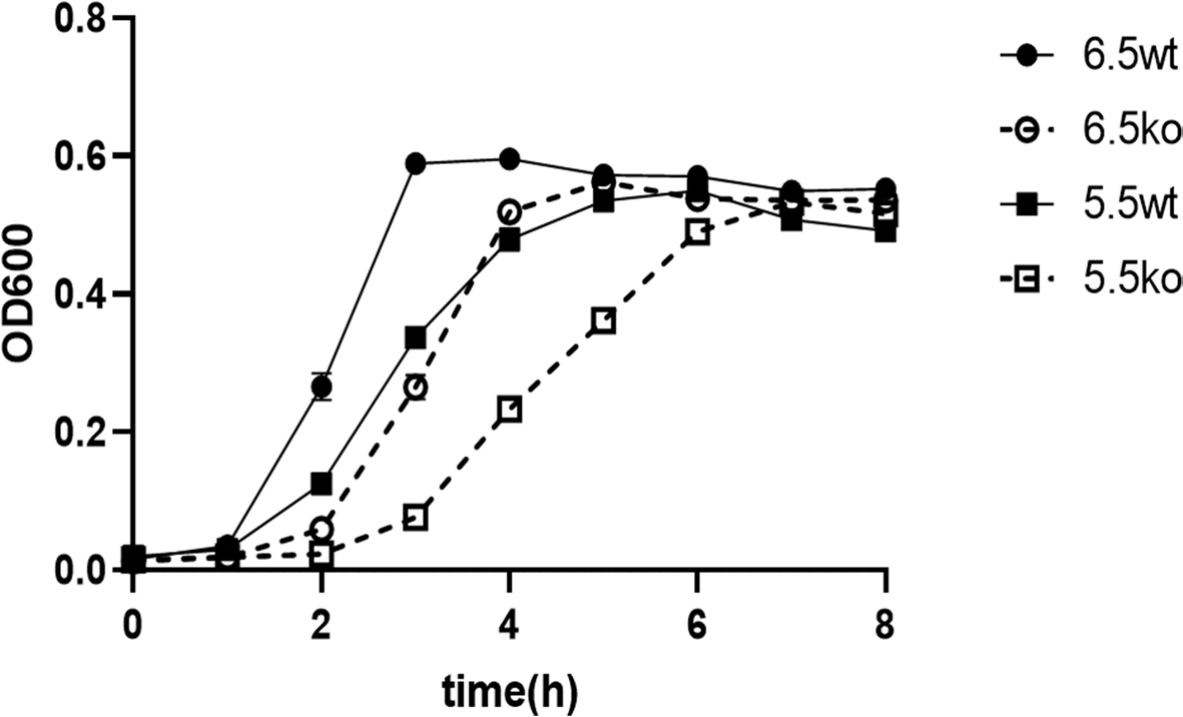
Growth curve of wild-type strain (wt) and *ccpA*-knockout strain (ko) of *S. bovis* S1 at different pH (pH 6.5 and pH 5.5) measured as optical density at 600 nm (OD 600). Values are the means (n = 3), with standard deviation indicated by vertical bars

Figure 2 shows the growth curve of *S. bovis* S1wild-type strain and *ccpA* deletion strain at different pH. When the pH was 6.5, the wild-type strain reached the stationary phase at 3 h and its OD value was 0.59, while the *ccpA*-deficient strain reached the stationary phase at 5 h and its OD value was 0.56. Under the pH of 5.5, the wild-type strain reached the stationary phase at 6 h and its OD value was 0.55, while the *ccpA*-deficient strain reached the stationary phase at 7 h and its OD value was 0.53.

Nonlinear fitting of the growth curves of *S. bovis* S1wild-type strain and *ccpA*-deficient strain at different pH were constructed using the logistic function model. Results showed that the R^2^ of the fitting equations were all above 0.99. The corresponding fitting parameters are shown in Table 2. There was a significant interaction between strain and pH for the growth lag period (λ) (*P* < 0.01) and maximum specific growth rate (μ_max_) (*P* < 0.05). Compared with the wild-type strain at pH 6.5, the μ_max_ of other groups were reduced (*P* < 0.05), and the *ccpA*-deficient strain at pH 5.5 had the lowest μ_max_ (*P* < 0.05). The λ values of *ccpA*-deficient strain at both pH were increased compared with those of the wild-type strain, and the *ccpA*-deficient strain at pH 5.5 had the highest λ (*P* < 0.01).

**Table 2:** The maximum specific growth rate and lag phase of wild-type strain and *ccpA*-knockout strain of *S. bovis* S1 at different pH based on the prediction from logistic function (*n* = 3)

Items^1^	Wild-type strain		*ccpA*-knockout strain		SEM^2^	*P-*value^3^		
	pH 6.5	pH 5.5	pH 6.5	pH 5.5		Strain	pH	Strain×pH
μ_max_	2.37^a^	1.58^b^	1.77^b^	1.29^c^	0.122	< 0.001	< 0.001	0.032
λ	0.74^c^	0.88^c^	1.17^b^	1.82^a^	0.128	< 0.001	< 0.001	0.002

^1^ μ_max_ means the maximum specific growth rate (h^−1^); λ means the growth lag period (h)

^2^ SEM, standard error of mean

^3^ Strain, strain effect; pH, pH effect; Strain × pH, the interaction between strain and pH

^a,b,c^ Means within a row with different superscripts differ significantly (*P* < 0.05)

### Organic acids production

Table 3 shows the organic acid production characteristics of *S. bovis* S1 wild-type strain and *ccpA* deletion strain at different pH. Results indicated that both strains produced lactic acid, formic acid, and acetic acid at different pH. Among them, lactic acid made up the largest proportion of organic acids followed by formic acid, with acetic acid being the least abundant. The interaction between strain and pH affected the concentration of total organic acids (*P* < 0.05) and acetic acid (*P* < 0.01), and the acetic acid molar percentage to total organic acids (*P* < 0.05). Compared with the wild-type strain at pH 6.5, the total organic acids concentrations of *ccpA*-deficient strain at pH 6.5 were increased, but those of both strains at pH 5.5 were decreased. The acetic acid concentration and its molar percentage to total organic acids of *ccpA*-deficient strain were higher than those of wild-type strain at both pH 6.5 and pH 5.5, with the highest at pH 6.5. The significant strain × pH interaction was not observed for lactic acid and formic acid concentration, and their molar percentages to total organic acids. Deletion of *ccpA* gene decreased the lactic acid concentration (37.38 vs 40.15 mM) and its molar percentage to total organic acids (73.83% vs 81.66%), while increased the formic acid concentration (8.07 vs 5.46 mM) and molar percentage (15.93% vs 11.10%) compared to the wild-type strain (*P* < 0.01). Strains at pH 5.5 had decreased concentrations of lactic acid (37.59 vs 39.94 mM; *P* < 0.01) and formic acid (6.51 vs 7.01 mM; *P* < 0.01), while an increased lactic acid molar percentage to total organic acids (78.06% vs 77.43%; *P* < 0.05) compared to those at pH 6.5.

**Table 3:** Effect of the absence of *ccpA* gene on organic acid production in *S. bovis* S1 at different pH (n = 3)

Items	Wild-type strain		*ccpA*-knockout strain		SEM^1^	*P-*value^2^		
	pH 6.5	pH 5.5	pH 6.5	pH 5.5		Strain	pH	Strain×pH
Lactic acid (mM)	41.10	39.20	38.78	35.98	0.561	< 0.001	< 0.001	0.107
Formic acid (mM)	5.63	5.29	8.40	7.74	0.404	< 0.001	0.002	0.200
Acetic acid (mM)	3.69^c^	3.43^d^	5.69^a^	4.70^b^	0.273	< 0.001	< 0.001	0.004
Total organic acids(mM)	50.42^b^	47.91^c^	52.87^a^	48.42^c^	0.603	0.001	< 0.001	0.014
Lactic acid (%)	81.51	81.81	73.35	74.31	1.190	< 0.001	0.049	0.260
Formic acid (%)	11.17	11.04	15.88	15.98	0.731	< 0.001	0.933	0.549
Acetic acid (%)	7.33^c^	7.15^c^	10.77^a^	9.71^b^	0.473	< 0.001	0.010	0.041

^1^ SEM, standard error of mean

^2^ Strain, strain effect; pH, pH effect; Strain × pH, the interaction between strain and pH

^a,b,c,d^ Means within a row with different superscripts differ significantly (*P* < 0.05)

### Enzyme activity and gene expression

Table 4 shows the enzyme activities of *S. bovis* S1 wild-type strain and *ccpA*-deficient strain at different pH. The interaction between strain and pH did not affect the activities of α-amylase and lactate dehydrogenase. Lower activities of α-amylase (32.74 vs 37.07 U/L) and lactate dehydrogenase (18.75 vs 22.06 U/L) were observed for *ccpA*-deficient strain compared to the wild-type strain (*P* < 0.01), and higher activities of these two enzymes (38.67 vs 31.14 U/L and 21.65 vs 19.16 U/L) were observed at pH 5.5 than at pH 6.5 (*P* < 0.01). There was a significant interaction between strain and pH for the activity of acetate kinase, which was the highest in the *ccpA*-deficient strain at pH 6.5 (*P* < 0.05).

**Table 4:** Effect of the absence of *ccpA* gene on enzymes activity in *S. bovis* S1 at different pH (*n* = 3)

Items	Wild-type strain		*ccpA*-knockout strain		SEM^1^	*P-*value^2^		
	pH 6.5	pH 5.5	pH 6.5	pH 5.5		Strain	pH	Strain×pH
Lactate dehydrogenase (U/L)	20.46	23.66	17.85	19.65	0.659	< 0.001	< 0.001	0.137
α-amylase (U/L)	33.87	40.28	28.41	37.06	1.345	< 0.001	< 0.001	0.096
Acetokinase (U/L)	2.34^c^	2.43^c^	3.06^a^	2.71^b^	0.089	< 0.001	0.101	0.013

^1^ SEM, standard error of mean

^2^ Strain, strain effect; pH, pH effect; Strain × pH, the interaction between strain and pH

^a,b,c^ Means within a row with different superscripts differ significantly (*P* < 0.05)

**Fig. 3:**
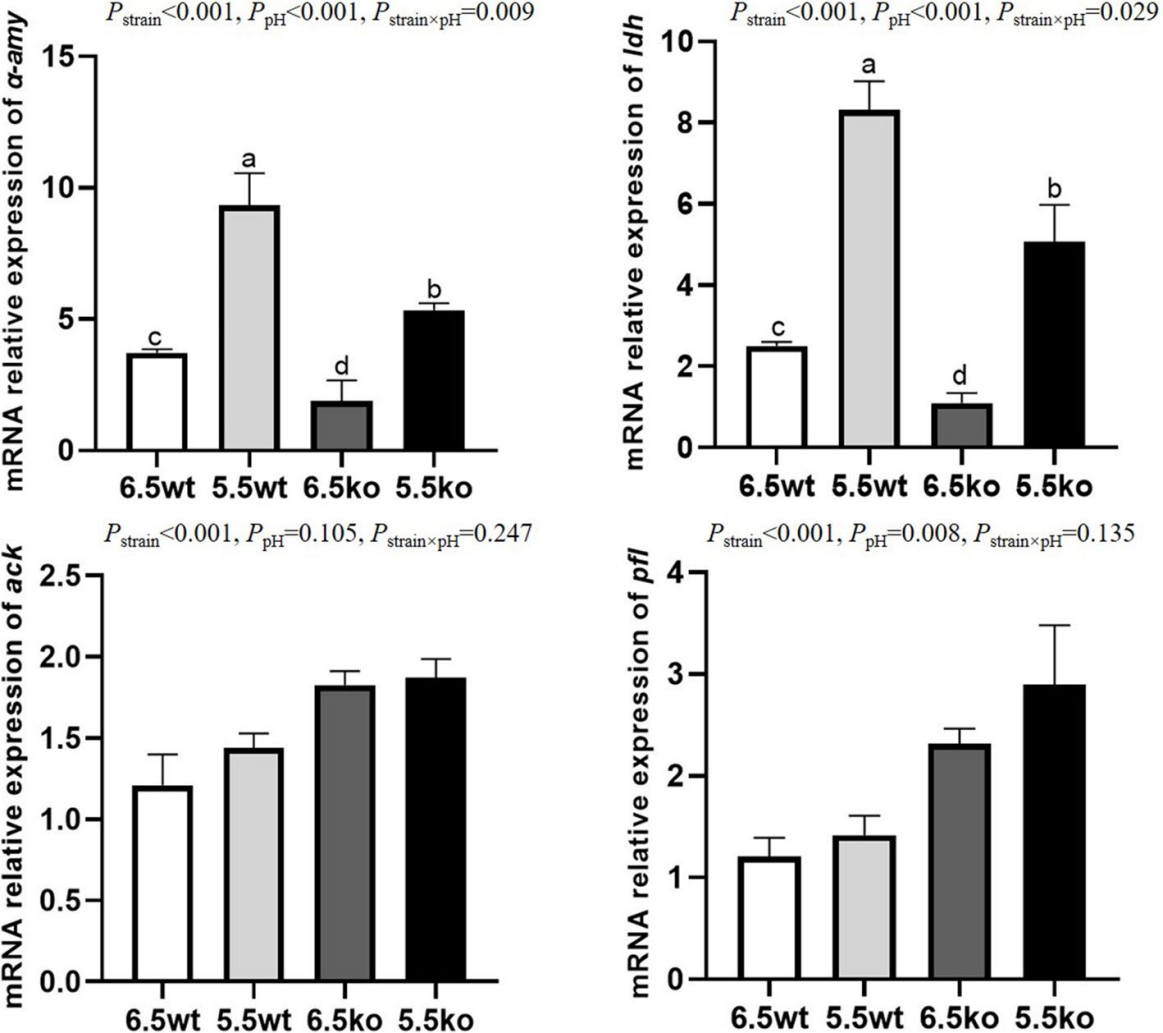
Relative mRNA expressions of *α-amy*, *ldh*, *ack* and *pfl* in wild-type strain (wt) and *ccpA*-knockout strain (ko) of *S.bovis* S1 at different pH. Values are the means (*n* = 3), with standard deviation indicated by vertical bars. *α-amy*, α-amylase; *ldh*, lactate dehydrogenase; *ack*, acetokinase; *pfl,* pyruvate formate lyase

Figure 3 shows results of the relative expressions of *α*-*amy*, *ldh*, *pfl*, and *ack* of *S. bovis* S1 wild-type strain and *ccpA*-deficient strain at different pH. The strain × pH interaction was significant for the relative expression of *ldh* (*P* < 0.05) and *α-amy* (*P* < 0.01), both of which were the highest in the wild-type strain at pH 5.5 and the lowest in the *ccpA*-deficient strain at pH 6.5. The interaction between strain and pH did not affect the relative expression of *pfl* and *ack.* The relative expressions of *pfl* and *ack* in the *ccpA*-deficient strain were increased compared to the wild-type strain (*P* < 0.01). The lower pH improved the relative expression of *pfl* (*P* < 0.01), while did not affect the relative expression of *ack.*

## Discussion

Ruminal pH generally fluctuates within a physiological range of about 5.5–7.0 in a 24-h period, which is driven by the amount of fermentable carbohydrate in each meal, innate buffering capacity of animals, and the absorption rate of organic acids [25, 26]. However, the rate of organic acids production is greater than absorption and the buffering capacity is also limited by inadequate salivary secretion when ruminants consume a large amount of rapidly fermentable (non-fiber) carbohydrates. As a result, ruminal pH will drop below its physiological level, which influences microbial composition and fermentation [3, 27]. It is worth noting that pH is an important environmental factor for bacteria, which not only affects the bacterial growth and fermentation rate, but also the final yield and purity of fermentation products [28]. In the rumen, the growth and organic acid fermentation pathways of most bacterial are affected by ruminal pH [27, 29], even for acid-tolerant species such as *S. bovis* and *Lactobacillus* [7].

*S. bovis* can grow in the pH range of 4.5–6.7 with the highest growth rate at pH 6.4 [6]. In this study, the maximum specific growth rate of *S. bovis* S1 wild-type strain was greater at pH 6.5 than pH 5.5, indicating that the low pH inhibited the growth of *S. bovis* S1. This finding is consistent with the characteristic of this strain found by Chen et al. [11], and *ccpA* deletion does not alter this characteristic. The absence of *ccpA*, at both pH 6.5 and 5.5, caused an extended lag phase and decreased maximum specific growth rate of *S. bovis* S1, which was consistent with the results obtained in other lactic acid-producing bacteria, such as *Lactobacillus bulgaricus* [21] and *Lactobacillus casei* [30]. However, the growth differences between the wild-type strain and *ccpA*-disrupted mutant of *S. bovis* 12 U1 have not been previously observed [12], which is inconsistent with present findings. This discrepancy may be attributed to the different growth and metabolic functions of strains isolated from different environments, even if they belong to the same species [31]. Besides, the significant interaction between strain and pH was observed for λ and μ_max_ in this study, which caused a lowest maximum specific growth rate and a longest lag phase in the *ccpA*-deficient strain at pH 5.5. The finding suggests a possible synergic effect between low pH and *ccpA* deletion on suppressing the growth of *S. bovis* S1, which is probably because that both deletion of *ccpA* and low pH could inhibit its growth, and the gene expression of *ccpA* in *S. bovis* S1 wild-type strain is influenced by the pH [11]. The results of present study imply that deletion *ccpA* gene would have a better effect on controlling the overgrowth of *S. bovis* S1 in rumen when ruminal pH is low, which need to be verified in the further study. We did not observe significant differences in maximum specific growth rate between wild-type strain at pH 5.5 and *ccpA*-disrupted mutant at pH 6.5, indicating that *ccpA* deletion inhibited the growth of *S. bovis* S1 only if the strain grown at the same pH condition.

The organic acid fermentation pattern of *S. bovis* is regulated by extracellular pH and growth rate [32, 33]. An earlier study found that at pH 6.7, lactic acid was the primary fermentation product when *S. bovis* JB1 was grown with a relatively fast growth rate, while it changed to formic acid, acetic acid and ethanol fermentation when the strain was grown slower in continuous culture [34]. As the pH dropped to 4.7, *S. bovis* JB1 mainly produced lactic acid even at slow growth rate, which is because the intracellular pH at this condition is similar to the optimal pH of *S. bovis* LDH [8, 34, 35]. However, the fermentation pattern of *S. bovis* S1 in the present study did not change when the extracellular pH dropped from 6.5 to 5.5, and the molar percentage of lactic acid is more than 80% of total organic acids at both pH, which is similar to the outcome previously observed in our lab [11]. The use of a batch culture and possibly insufficiently low extracellular pH of the current study may provide some explanation for the unaltered fermentation pattern of *S. bovis* S1 as extracellular pH dropped. Although the fermentation pattern was not affected by the extracellular pH in the present study, low pH reduced the final concentrations of lactic acid, formic acid and total organic acids of *S. bovis* S1 wild-type strain. Similarly, this pattern of changes was also found after *ccpA* knockout. The production of organic acids is associated with the specific activity and amount of enzymes. In *Streptococcal*, the LDH has a requirement for FDP [36], and the concentration of FDP produced by *S. bovis* S1 at pH 5.5 is lower than pH 6.5 [11], which may be the reason for the decreased lactic acid production of this study even though the activity of LDH and the *ldh* gene expression in both strains were significantly higher at pH 5.5 than pH 6.5.

Inactivation of the *ccpA* gene significantly decreased lactic acid production, and increased the production of formic acid and acetic acid of *S. bovis* S1 growing at both pH 6.5 and pH 5.5. The findings are consistent with the results of Asanuma et al. [12]. Subsequently, we further analyzed the activity and gene expression of related enzymes involved in the organic acids production pathway. The activity and gene expression of enzyme for lactic acid production (LDH) were reduced, and enzymes for formic acid and acetic acid production (PFL and ACK) were increased in the mutant strain compared to the wild-type strain. This observation may explain the altered production of organic acids in *ccpA*-disrupted mutants. Similar results were found in other lactic acid bacteria. For example, Asanuma et al. [12] found that the ratio of formic acid to lactic acid in *S. bovis* 12 U1 increased significantly after *ccpA* gene deletion, and this change was directly associated with changes in related enzyme activities and gene expression. The transcription of the *las* operon encoding phosphofructokinase (*pfk*), pyruvate kinase (*pk*), and lactate dehydrogenase (*ldh*) in *L. lactis* was reduced by 75% in the *ccpA* deletion mutant strain compared to the wild-type strain, which increased ethanol and acetic acid and decreased lactic acid production [37]. Moreover, the activities of LDH, PK, and PFK in *L. bulgaricus* were significantly reduced after *ccpA* gene deletion, which resulted in reduced lactic acid yield [21]. In *S. mutans*, CcpA has been demonstrated to regulate the transcription of *ackA* (acetate kinase A) and *pta* (phosphotransacetylase) in response to pH [22]. In the present study, there was a significant interaction between strain and pH for the concentration of total organic acids and acetic acid production, which led to the highest total organic acids and acetic acid production in *ccpA*-disrupted mutant at pH 6.5. Moreover, a significant interaction between strain and pH was observed for the activity of acetate kinase and gene expressions of *ldh* and *α-amy*. The interaction effect between pH and *ccpA* gene deletion on these indicators is possibly caused by the fact that the expression of *ccpA* gene is regulated by extracellular pH in *S. bovis* S1 [11]. However, further experiment will be needed to verify this speculation.

## Conclusion

Low pH inhibits the growth of *S. bovis* S1, and lowers the production of organic acids but does not alter the production pattern despite the increased activities of α-amylase and LDH. The deletion of *ccpA* gene inhibits the growth of *S. bovis* S1 and regulates the organic acid fermentation pattern towards lower lactic acid and higher formic acid and acetic acid, suggesting that CcpA is probably involved in the carbon metabolism of *S. bovis* S1. Moreover, there seems to be an additive effect between pH and *ccpA* deletion on regulating the growth and organic acids production of *S. bovis* S1. The mechanism underlying this interaction remains to be clarified.

## Supplementary Information

**Additional file 1.**

## Data Availability

The sequence data during the current study are available in the [figshare] repository, [10.6084/m9.figshare.14779206].

## Abbreviations

*α-amy*: α-amylase
*ack*: Acetic acid kinase
CcpA: Catabolite control protein A
*cre*: Catabolite response element
FDP: Fructose-1,6-diphosphate
HPr: Heat-stable protein
*ldh*: Lactate dehydrogenase
OD: Optical density
*pfl*: Pyruvate formate lyase
*pta*: Phosphotransacetylase
SEM: Standard error of mean
