# Supplementary figures and images for: Regulation of CcpA on the growth and organic acid production characteristics of ruminal Streptococcus bovis at different pH
Source: BMC Microbiol. 2021 Dec 15;21:344. doi: 10.1186/s12866-021-02404-x (PMC8672513; doi:10.1186/s12866-021-02404-x)

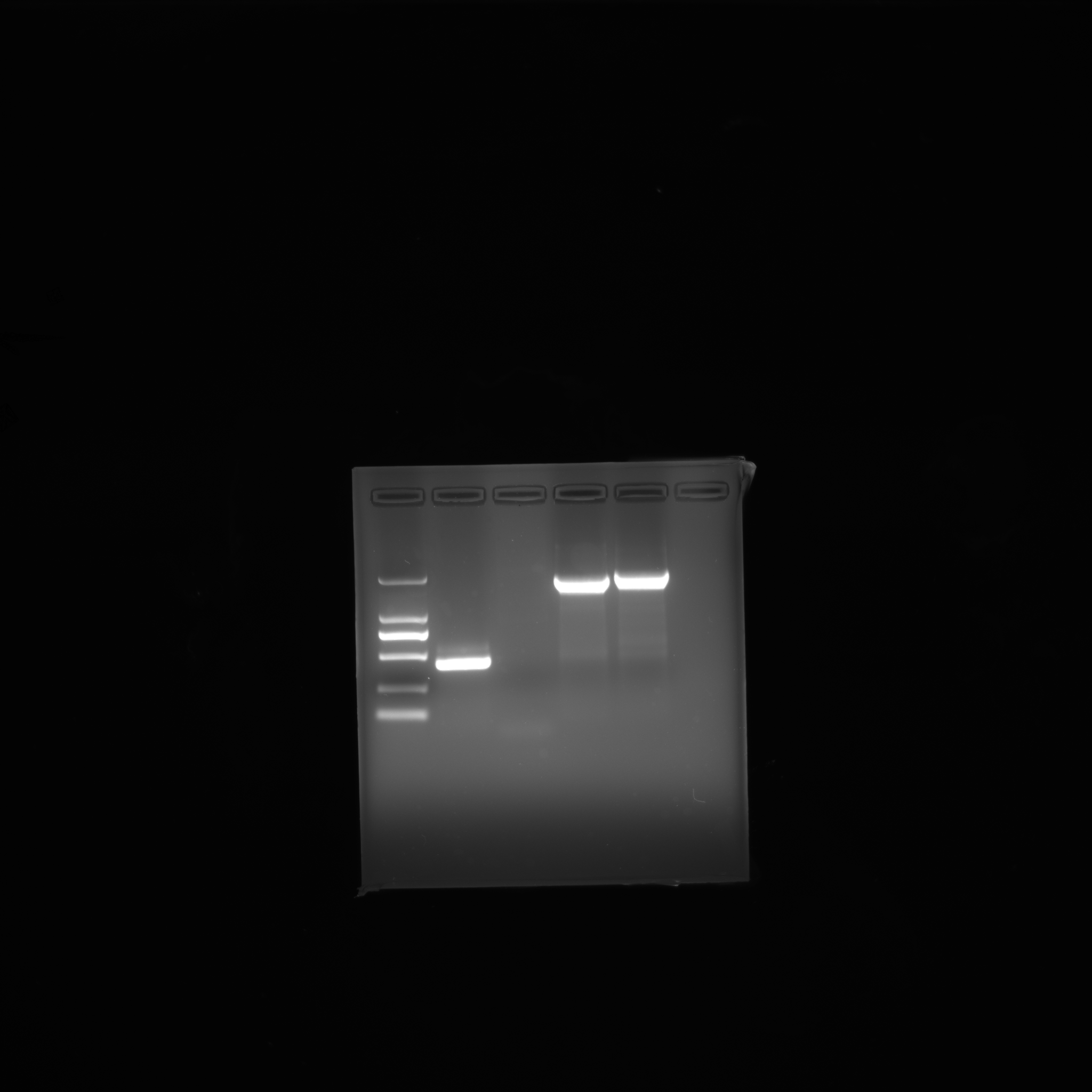

Supplement: Supplementary file 1 — Additional file 1. [file 12866_2021_2404_MOESM1_ESM.tif]
